# Severe Heterotopic Ossification in the Skeletal Muscle and Endothelial Cells Recruitment to Chondrogenesis Are Enhanced by Monocyte/Macrophage Depletion

**DOI:** 10.3389/fimmu.2019.01640

**Published:** 2019-07-19

**Authors:** Mario Tirone, Anna Giovenzana, Arianna Vallone, Paola Zordan, Martina Sormani, Pier Andrea Nicolosi, Raffaela Meneveri, Carmen Rosaria Gigliotti, Antonello E. Spinelli, Renata Bocciardi, Roberto Ravazzolo, Ingrid Cifola, Silvia Brunelli

**Affiliations:** ^1^School of Medicine and Surgery, University of Milano-Bicocca, Monza, Italy; ^2^Division of Immunology, Transplantation and Infectious Diseases, San Raffaele Scientific Institute, Milan, Italy; ^3^Division of Regenerative Medicine, San Raffaele Scientific Institute, Milan, Italy; ^4^Medical Physics Department, San Raffaele Scientific Institute, Milan, Italy; ^5^Centre for Experimental Imaging, San Raffaele Scientific Institute, Milan, Italy; ^6^Department of Neurosciences, Rehabilitation, Ophthalmology, Genetics, Maternal and Child Health, Università degli Studi di Genova, Genova, Italy; ^7^U.O.C. Genetica Medica, IRCCS Istituto Giannina Gaslini, Genova, Italy; ^8^Institute for Biomedical Technologies (ITB), National Research Council (CNR), Milan, Italy

**Keywords:** macrophage, endothelial cell (EC), heterotopic ossification (HO), EndoMT, endothelial progenitors cells, RNASeq and NGS data analysis, micro-computerized tomography (μCT) analysis

## Abstract

Altered macrophage infiltration upon tissue damage results in inadequate healing due to inappropriate remodeling and stem cell recruitment and differentiation. We investigated *in vivo* whether cells of endothelial origin phenotypically change upon heterotopic ossification induction and whether infiltration of innate immunity cells influences their commitment and alters the ectopic bone formation. Liposome-encapsulated clodronate was used to assess macrophage impact on endothelial cells in the skeletal muscle upon acute damage in the ECs specific lineage-tracing Cdh5CreER^T2^:R26REYFP/dtTomato transgenic mice. Macrophage depletion in the injured skeletal muscle partially shifts the fate of ECs toward endochondral differentiation. Upon ectopic stimulation of BMP signaling, monocyte depletion leads to an enhanced contribution of ECs chondrogenesis and to ectopic bone formation, with increased bone volume and density, that is reversed by ACVR1/SMAD pathway inhibitor dipyridamole. This suggests that macrophages contribute to preserve endothelial fate and to limit the bone lesion in a BMP/injury-induced mouse model of heterotopic ossification. Therefore, alterations of the macrophage-endothelial axis may represent a novel target for molecular intervention in heterotopic ossification.

## Introduction

The regenerating skeletal muscle niche is a complex environment where distinct cell populations play crucial and non-redundant roles. Progenitors in the muscle comprise the satellite cells, which are quiescent stem cells that, once activated, are the primary myogenic cells responsible for skeletal muscle regeneration (1). Vascular progenitors and interstitial cells, such as fibroadipogenic precursors (FAPs) and PW1^+^/Pax7^−^ interstitial progenitor cells (PICs), contribute to muscle regeneration in several ways. They induce the formation of the new capillary network to provide nutrients and oxygenation, they directly differentiate to muscle fibers (2, 3) and produce growth factors and other soluble signals essential for proper stem cell activation, myogenic differentiation and reconstitution of the contractile apparatus (4–7). Disruption of vessel assembly and jeopardized angiogenesis concur to muscle wasting (8, 9).

Increasing evidences support the hypothesis that these interactions and processes need to be orchestrated and coordinated by the cells of the immune system, in particular macrophages (MPs), that infiltrate the muscle immediately after the initial tissue injury and necrosis and release several cytokines (10, 11). The two main macrophage populations, i.e., the classically activated inflammatory MPs and the alternatively activated MPs, play a sequential role to set the pace of muscle regeneration upon acute injury (11–14). Failure of macrophage recruitment or altered polarization results in impaired tissue regeneration and fibrosis, as described in several chronic pathological conditions (10, 15). Furthermore, we have previously demonstrated that a proper macrophage recruitment in muscle after an acute sterile damage is also essential for maintaining a correct angiogenic program and prevent the endothelial contribution to scar formation (16). This seems to occur through a complex biological process, referred to as endothelial to mesenchymal transition (EndoMT). EndoMT involves loss of endothelial cell (EC) identity in favor of a multipotent mesenchymal phenotype, which often contributes to exacerbate the severity of many different fibrotic disorders not only in the muscle but also in kidney, liver and heart (17–19). Many recent studies have suggested that ECs have the potential to differentiate along other mesenchymal derived lineages, including chondrocytes and osteogenic precursors (20–24).

Extra-skeletal osteogenesis is a sporadic event with serious clinical consequences. Defined as heterotopic ossification (HO), it involves the development of an endochondral bone in soft tissues due to fracture complication, tissue injury, neurological trauma or genetic defects, such as fibrodysplasia ossificans progressiva (FOP, OMIM 135100) (25, 26). FOP arises from gain-of-function mutations in the bone morphogenetic protein (BMP) type I receptor gene *ACVR1* (alias *ALK2*), resulting in aberrant activation of the BMP signaling pathway and acquired sensitivity to unconventional ligands of the mutated receptor (27–29). Deregulation of the BMP pathway is also a feature of acquired HO (30, 31). Lineage tracing studies have suggested that several different cell populations may contribute to the ectopic bone formation both in genetic and pharmacological models of FOP, amongst which are ECs (32–37). Nonetheless, a definitive endorsement of ECs as contributing to heterotopic ossification remains controversial, partially due to the modest specificity or efficiency of the lineage tracing tools used.

In this study, we rely on a specific and efficient *in vivo* endothelial genetic tracing mouse model to unambiguously demonstrate the contribution of EC-derived cells to BMP-dependent ectopic chondro-ossification and to determine that MPs play a non-redundant role in controlling this process.

## Results

### EC-Derived Cells in the Regenerating Skeletal Muscle Acquire an EndoMT/Chondrogenic Gene Expression Signature Upon Monocyte/Macrophage Depletion

**Figure 1:**
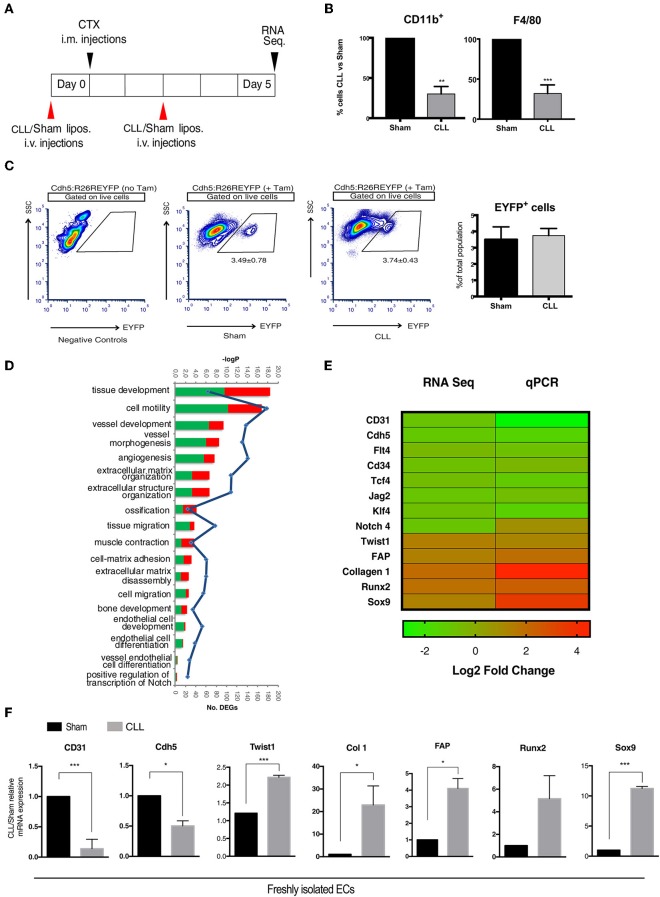
Gene expression profiling of EC-derived cells. **(A)** Experimental scheme of muscle acute injury and macrophage depletion (left panel). **(B)** Estimation of the percentage of Cd11b^+^ and F4/80^+^ cells (macrophages) in the blood of clodronate (CLL) vs. control (Sham) treated mice (right panel), 5 days after injury. Bars represent mean ± SEM. **p ≤ 0.01; ***≤0.001, *n* = 4. **(C)** EYFP^+^ gating strategy used for FACS analysis of single cell suspensions derived from CLL or Sham Cdh5-CreER^T2^:R26R-EYFP mice. Plots are representative of at least three independent experiments. Graph on the right shows the quantification of the percentage of EYFP^+^ sorted cells (EC derived cells). **(D)** Gene Ontology (GO) Biological Process terms enriched by differentially expressed genes (DEGs) found in CLL vs. control samples. Number of up- and down-regulated DEGs associated to each term are shown **as red and green** bars, respectively. All the enrichments shown are statistically significant (**blue line** indicates significance expressed as -log Pvalue). **(E)** Heatmap showing fold change in the expression of selected genes differentially expressed between EC derived cells isolated from muscle of CLL- and Sham-treated Cdh5-CreER^T2^:R26R-EYFP mice, as assessed by RNAseq and qRT-PCR. **(F)** qRT-PCR expression assays for a panel of selected genes. Values are expressed as fold changes relative to Sham mRNA expression and normalized on the housekeeping (cyclophilln A). Bars represent mean ± SEM. *p ≤ 0.05; **≤0.01; ***≤0.001, *n* = 4.

To study how the interplay between immune system and ECs may contribute to trauma-induced heterotopic bone formation, we chose a mouse model of severe muscle acute injury, where macrophage infiltration is compromised. In this model, we have previously shown that EndoMT and fibrosis are induced (16). We generated double transgenic Cdh5CreER^T2^:R26REYFP mice by crossing Cdh5CreER^T2^ transgenics with R26REYFP reporters. By inducing the Cre activity at perinatal stages, this mouse model guarantees very high efficiency and specificity of the original labeling of endovascular progenitors and mature ECs, thus excluding marking of any cell of mesenchymal or hematopoietic lineages (16, 32, 38–40). In this model, we have targeted MPs by intravenously injecting liposomes containing clodronate (CLL) or PBS as control (Sham) 1 day before and every other day after acute damage induction by cardiotoxin (CTX) injection, and we freshly sorted EYFP^+^ ECs from muscle of CLL- and Sham-treated mice 5 days after CTX (Figure 1A), a time where we did not see yet major changes in the EC-derived population, only a slight delay in capillary re-organization (16). The monocyte/macrophage depletion efficiency, as assessed by measuring the fraction of CD11b^+^ and F4/80^+^ cells in peripheral blood of CLL- and Sham- treated mice by flow cytometric analysis, was comparable to our previous studies (Figure 1B, Supplementary Figures S1A,B) (16, 40). This depletion *per se* did not affect the number of EC-derived cells that could be retrieved (Figure 1C). In addition, FACS analysis on EYFP+ sorted cells revealed that in both Sham and Cll treated mice almost all the EYFP^+^ cells express the endothelial marker CD31 (95,23 ± 2,56 and 96,56 ± 2,02, *n* = 3). This is in agreement with the finding that all EC (mature and progenitors) express Cdh5 and CD31, the latter being expressed at lower levels in endothelial progenitors (38, 39) (Supplementary Figures S2A,B). In both conditions, few cells co-expressed CD31 and PDGFR-β, a PDGB receptor expressed in pericyte but also in lymphatic and embryonic ECs and in endothelial progenitors cells (32, 38, 41, 42). α7-integrin, marker of mesenchymal and satellite cells, was not significantly expressed (43) (Supplementary Figures S2A,B). These finding indicated that our sorting strategy allowed us to obtain a population of EC-derived cells devoid of significant contamination, suitable for further bulk transcriptomic analysis.

We next characterized by next-generation sequencing (RNA-seq) analysis the transcriptome profile of genetically labeled isolated cells of endothelial origin (EYFP^+^ ECs). As a result, we identified 1,399 genes that, just at 5 days after injury, show a statistically significant differential expression (DEGs) between CLL- and Sham-treated samples (Supplementary Table S1). The Gene Ontology enrichment analysis showed, as expected, that these DEGs are mainly involved in biological processes related to EndoMT and mesenchymal-fibrogenic features (Figure 1D). qRT-PCR analysis showed in CLL treated mice (Figures 1E,F), a downregulation of genes encoding for endothelial surface proteins (CD31, Cdh5, CD34), for signaling molecules (Notch4, Flt4, Jag2) (32), and transcription factors (TCF4 and KLF4) (44, 45) involved in endothelial differentiation. Conversely EYFP^+^ cells from CLL treated mice upregulated genes coding for mesenchymal/fibrogenic markers such as Collagen 1 and Fibroblast activating protein (FAP) and for transcription factors involved in EndoMT such as TWIST1 (19). Notably, in cells from CLL treated mice we also found an enrichment for genes involved in ossification and bone development processes (Figure 1D). EYFP^+^ cells from CLL mice showed upregulation of Sox9 and Runx2 (Figures 1E,F), master genes of endochondral differentiation (46). These data suggest that macrophage depletion not only promote EndoMT as shown before, thus increasing endothelial plasticity (16, 40) but may also enhance their chondro-osteogenic potential.

### EC-Derived Cells Contribute to BMP-Induced Chondrogenesis

**Figure 2:**
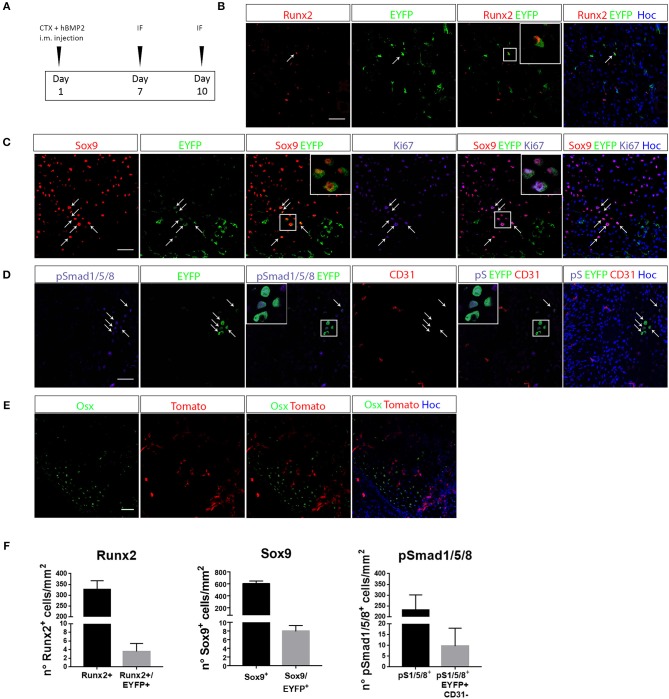
EC-derived cells contribute to the development of ectopic bone. **(A)** Experimental scheme of BMP2-mediated bone induction. **(B–D)** IF of muscle section from quadriceps of Cdh5-CreER^T2^:R26R-EYFP mice at 7 days after bone induction and stained with Runx2 and EYFP antibodies **(B)**, Sox9, EYFP and Ki67 antibodies **(C)** or pSMAD1/5/8, EYFP and CD31 antibodies **(D)**. Right panels showed merged images, where nuclei are stained with DAPI (blue). In B arrows indicate cells that co-express both Runx2 and EYFP. Inset show magnification (3 ×) of double positive cells. In **C**, arrows indicate cells that co-express both Sox9 and EYFP, insets show magnification (3 ×) of cells expressing Sox9 and EYFP, or Sox9, EYFP, and Ki67. In **D** arrows and insets with magnification show pSMAD1/5/8^+^ cells of endothelial origin (EYFP^+^) that have lost CD31 endothelial marker. **(E)** IF of muscle section from quadriceps of Cdh5-CreER^T2^:dtTomato mice at 10 days after bone induction and stained with Osterix (Osx) antibody. Right panel shows merged images, where nuclei are stained with DAPI (blue). No double positive dtTomato^+^/Osx^+^ were detected in this condition. Magnification 40 ×. Scale bars for all panels, 50 μm. **(F)** Quantification of the number of total number of Runx2^+^, Sox9^+^ or Smad1/5/8^+^ cells/mm^2^ and the number of EYFP double positive cells (Sox9^+^/EYFP^+^, Runx2^+^/EYFP^+^, pSmad1/5/8^+^/CD31^+^/EYFP^+^).

To verify whether EC derived cells really contribute to the process of ectopic chondro-ossification, we first induced the formation of cartilage and bone in the muscle of Cdh5CreER^T2^:R26REYFP or Cdh5CreER^T2^:tdTomato mice, by intramuscular injection of rhBMP2, together with CTX, as described in Cappato et al. (47) (Figure 2A). This HO model is a reasonable approximation for HO in humans since it causes a highly reproducible sequence of molecular signals and histological events, ultimately leading to the emergence of endochondral bone, thus mimicking the histological changes seen in both acquired HO and FOP (31, 36). After 7 days, we could detect the presence of EYFP^+^ ECs co-expressing the chondrocyte markers Sox9 and Runx2 (Figures 2B,C,F, Supplementary Table S2). Interestingly, many EYFP^+^/Sox9^+^ cells expressed Ki67, indicating that they are still proliferating and suggesting that Cdh5-derived cells do not transdifferentiate directly to chondrocytes, but cell cycle entry may be involved (Figure 2C). A number of EYFP^+^ cells also express pSMAD1/-/5-/8 (Figures 2D,F), demonstrating they have activated the chondrogenic pathway and display high BMP signaling, suggestive of BMP type I receptor kinase activation by rhBMP2. Interestingly, some EYFP^+^ pSMAD^+^ cells did not express anymore the endothelial marker CD31 (Figure 2D). On the other hand, after 10 days we could not detect EC-derived cells expressing the osteogenic master gene Osterix (Osx) (Figure 2E), showing that in these conditions EC-derived cells did not contribute to ectopic ossification. These results are indicative that upon enhancement of BMP signaling, coupled with a muscle injury, ECs show the potential to contribute to the formation of ectopic cartilage.

### Macrophage Depletion Exacerbates BMP-Induced Ectopic Ossification and Conversion of EC-Derived Cells to Chondrocytes

**Figure 3:**
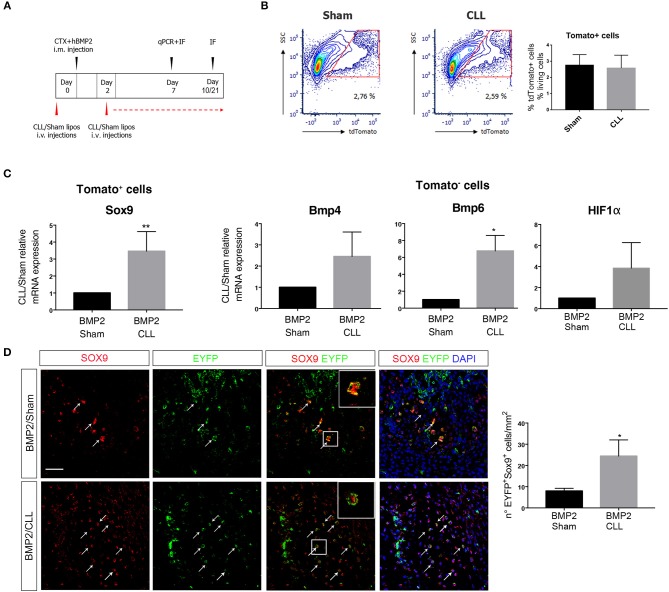
Macrophage depletion increases EC-derived cells contribution to endochondral differentiation. **(A)** Experimental scheme showing BMP2-mediated bone induction, clodronate (CLL) and Sham liposome treatments, and sacrifice time-points. **(B)** Gating of tdTomato^+^ sorted cells from Cdh5-CreER^T2^:dtTomato mice quadriceps at 7 days after bone induction (left). On the right: graph showing the median percentage of dtTomato^+^ cells among the number of living cells (gated on physical parameters) in Sham and CLL-treated mice. Bars represent mean ± SEM (*n* = 4). **(C)** qRT-PCR expression of Sox9 in dtTomato^−^ cells (left) and of Bmp4 and Bmp6 in dtTomato^+^ cells (right), isolated from CLL or Sham Cdh5-CreER^T2^:dtTomato mice quadriceps at 7 days after bone induction. Data are expressed as fold changes relative to Sham mRNA expression and normalized on the housekeeping (cyclophillin A and/or 28S) (left). Bars represent mean ± SEM. *p ≤ 0.05; **≤0.01 (*n* = 4). **(D)** Sections of quadriceps of CLL and Sham Cdh5-CreER^T2^:R26R-EYFP mice at 7 days after HO induction. Panels show IF images of muscle sections stained with Sox9 and EYFP antibodies. Right panels showed merged images, where nuclei are stained with DAPI (blue). Scale bar 50 μM. Panel magnification 40 ×. Arrows and inset with magnification (3 ×) indicate cells that express both markers. Graphs on the right represent the number of Sox9^+^EYFP^+^ cells/mm^2^. Bars represent mean ± SEM. *p ≤ 0.05 (*n* = 4).

Infiltrating MPs prevent EndoMT upon acute muscle damage (16) and, as demonstrated above, macrophage depletion *per se* is sufficient to induce the transcriptional upregulation of the chondrocyte markers Sox9 and Runx2 in EC-derived cells (Figure 1D). In contrast previous studies reported that depletion of circulating phagocytes decrease ossification in transgenic FOP end neurological HO (48, 49). To explore and further clarify the role of infiltrating MPs during BMP-induced HO, we experimentally depleted phagocytes by intravenously injecting liposomes containing CLL (or PBS as control, Sham) every 2 days in BMP/CTX-treated Cdh5CreER^T2^:R26REYFP or Cdh5CreER^T2^:tdTomato mice (Figure 3A). In this condition as well, Cd11b^+^ and F4/80^+^ cell count in peripheral blood was reduced in CLL- vs. Sham-treated mice (Supplementary Figures S3A,B). Importantly, we showed that this treatment led to a significant decrease in the number of F4/80^+^ MPs that infiltrate the muscle (Supplementary Figures S4A–C), thus indicating that phagocyte targeting was really effective on the local population. In particular we observed a significant decrease in the number of F4/80^+^ macrophages also expressing the specific markers of alternative activation CD163 (Supplementary Figures S4A,C) and CD206 (Supplementary Figures S4B,C).

We first focused on the fate of EC-derived cells at early stages of chondro-ossification, by freshly isolating them from the muscle 7 days after BMP/CTX treatment (Figure 3B). dtTomato^+^ EC-derived cells from CLL-treated mice showed an increased expression of Sox9 gene with respect to control samples (Figure 3C). On the other hand, the dtTomato^−^ fraction in CLL-treated mice showed the upregulation of Bmp4 and Bmp6 gene expression, suggesting that MPs depletion leads to amplification of BMP signaling in the muscle (Figure 3C). This is consistent with an increased hypoxic environment as suggested by trend in α the upregulation of HIFa transcripts (Figure 3C) and in agreement with previous studies (50).

Immunofluorescence (IF) analysis confirmed that, 7 days after BMP/CTX injection, muscle from CLL mice contains an increased number of EC-derived cells (EYFP^+^ or dtTomato^+^) expressing Sox9 protein (Figure 3D).

Only in this condition we also observed that, 10 days after BMP/CTX, few EC-derived cells express Osx (Supplementary Figure S5A), indicating that some of these cells have the potential to enter an osteogenic fate. After 21 days some EC-derived cells also co-localize with the extracellular marker of mature osteoblast Osteocalcin (Supplementary Figures S5B,C).

**Figure 4:**
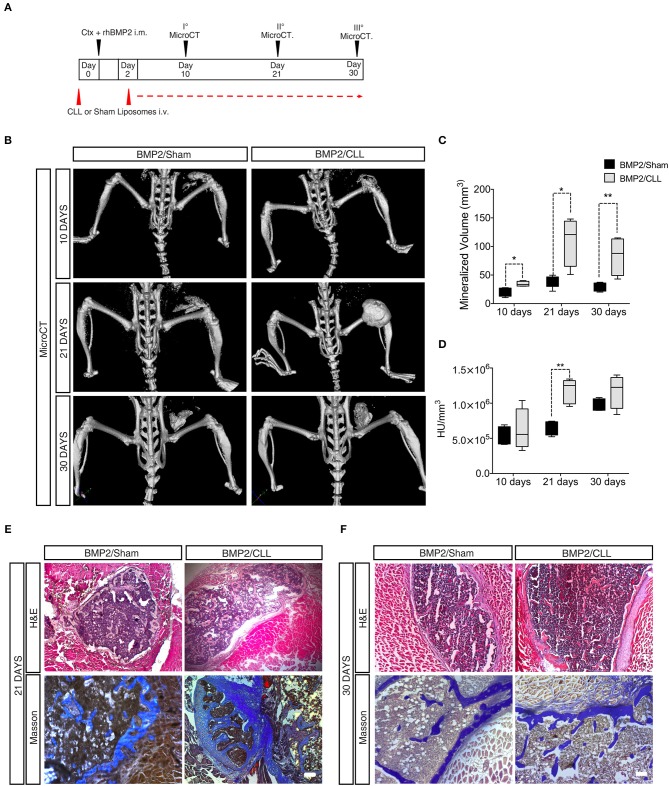
Macrophage depletion increases BMP2 mediated HO. **(A)** Experimental scheme showing BMP2-mediated bone induction, clodronate (CLL) and Sham liposome treatments, and micro-computerized tomography (μCT) time-points. **(B)** μCT scans of CLL or Sham Cdh5-CreER^T2^:tdTomato mice at 10, 21, and 30 days after bone induction. **(C)** Quantification of the mineralized ossicle volume (mm^3^). *n* = 4 mice. Box and whisker plots represent mean and min to max values. *p ≤ 0.05; **≤0.01. **(D)** Quantification of the ectopic bone density (HU/mm^3^). *n* = 4 mice. Box and whisker plot represent mean and min to max values. **p < 0.01. HU, Hounsfield unit. **(E,F)** H&E and Masson Trichrome staining of muscle section showing lesions in Sham or CLL Cdh5-CreER^T2^: tdTomato mice at 21 and 30 days after bone induction. Scale bar 50 μm.

*In vivo* micro-computerized tomography (μCT) scan was carried out on both CLL- and Sham-treated mice to assess the progression of ossification and any other effect on the normal skeletal structure at later stages (10, 21, and 30 days after BMP/CTX treatment) (Figure 4A). After 10 days, we could already detect significant differences in HO in CLL vs. control mice (measured as mineralized volume, mm^3^), although not in bone density (HU/mm^3^) (Figures 4C,D). Twenty one days after bone induction μCT scans showed a significant increase in both HO volume and bone density in CLL vs. control mice (Figures 4C,D, Supplementary Movies S1, S2). The significant difference in HO volume was maintained also at 30 days after treatment. Bone density was increased in CLL mice at 30 days, but with higher variability (Figures 4B,D). These results were confirmed by histochemical analysis using hematoxylin and eosin (H&E) and Masson staining, which revealed that, at both 21 and 30 days after HO triggering, the ectopic bone area appeared greater in CLL- vs. Sham-treated mice (Figures 4E,F). This implies that proper immune cells infiltration from the beginning of HO triggering is able to limit the extent of the ectopic bone lesions. In addition, *in vivo* μCT imaging also allowed us to show that only in CLL-treated mice there was an increase of fluorescence signal in the area of ectopic ossification at 10 and 21 days after BMP-induction, reflecting an increased recruitment of EC-derived cells upon macrophage depletion (Supplementary Movie S3), in agreement with previous studies showing that circulating ECs are recruited to the wound/HO site and undergo EndoMT (51).

**Figure 5:**
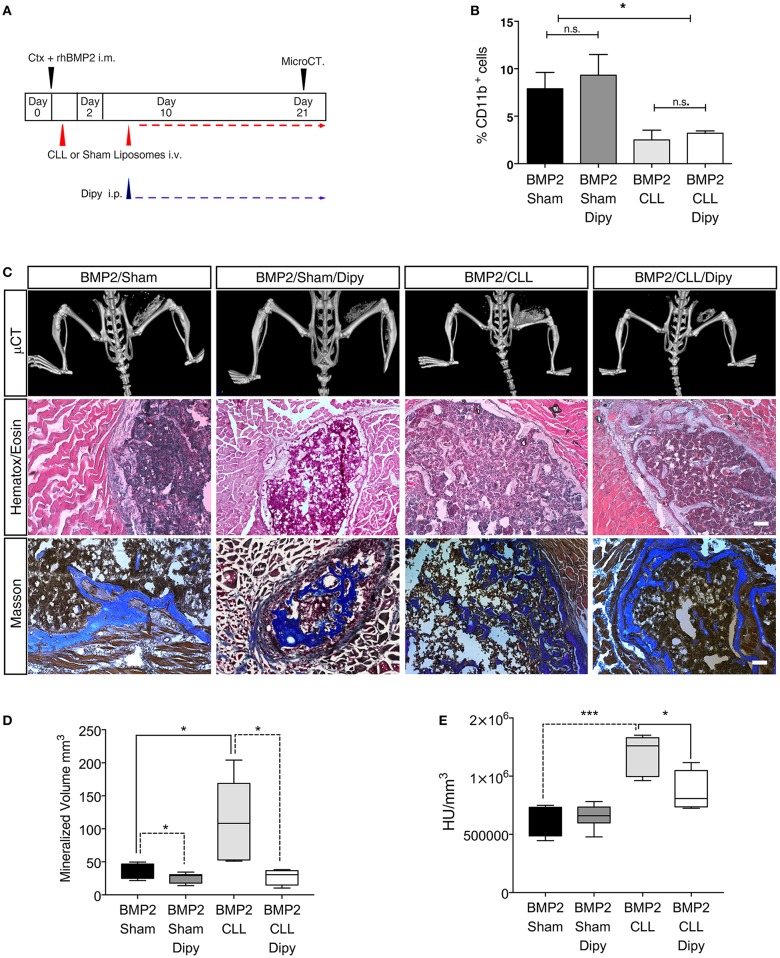
Dipyridamole decreases HO induced by macrophage depletion. **(A)** Experimental scheme showing BMP2-mediated bone induction, clodronate (CLL) and Sham liposome treatments, dipyridamole administration and micro-computerized tomography (μCT) time-points. **(B)** Graph representing the percentage of Cd11b^+^ cells after Sham, Sham/dipyridamole (Sham+Dipy), CLL or CLL/dipyridamole (CLL+Dipy) treatment. *n* = 5 mice. Bars represent mean ± SEM. **(C)** μCT scans of Cdh5-CreER^T2^:tdTomato mice treated with CLL (BMP2/CLL) or CLL/dipyridamole (BMP2/CLL/Dipy), PBS (BMP2/Sham) or PBS/dipyridamole (BMP2/Sham/Dipy) at 21 days after bone induction. Lower panels show representative images of bone lesions in muscle sections after H&E and Masson Trichrome staining. Scale bar 50 μm. **(D)** Quantification of the mineralized ossicle volume and **(E)** of the ectopic bone lesion density (HU/mm^3^) (right). Box and whisker plots represent mean and min to max values. *p ≤ 0.05; ***≤0.001 (*n* = 5).

Finally, we wanted to assess whether we could prevent the exacerbated bone formation developed upon macrophage depletion. Since altering the population of infiltrating macrophages led to an amplification of BMP signaling, and the targeting of this pathway has been suggested as a potential therapy for HO (52) we decided to treat Sham- or CLL-treated mice with dipyridamole, a drug that has an inhibitory effect on the whole SMAD-dependent BMP signaling pathway and partially inhibits the process of BMP-triggered HO (47) (Figure 5A). We first verified that dipyridamole did not lead to a differential CD11b^+^ monocyte depletion in CLL mice (Figure 5B). We therefore compared the extent of HO in Sham-, Sham/dipyridamole-, CLL-, and CLL/dipyridamole-treated mice by μCT and histochemistry (Figure 5C), and found that after 21 days dipyridamole was able to rescue HO in CLL mice (and in Sham treated mice as also previously reported) (47), reducing the mineralized bone volume and bone density to levels comparable to those of Sham mice (Figures 5D,E).

## Discussion

Heterotopic ossification (HO) is a pathological condition where extra-skeletal bone forms in soft tissues due to extreme trauma or genetic defects. Induction of ectopic bone formation is highly destructive. Therefore, its prevention has become an important focus of research, particularly with regards to pre- and post-operative preventive care in acquired heterotopic ossification and in fibrodysplasia ossificans progressiva (FOP) patients. Of particular relevance is the research aiming to identify the pathophysiological mechanisms of HO, thus eventually contributing to the development of new and targeted treatment options.

FOP is caused by mutations of the *ACVR1* gene, encoding the ALK2 bone morphogenetic protein (BMP) type 1 receptor, and the consequent dysregulation of the BMP/Activin/TGF-ß family ligand signaling is a shared property of both genetic and acquired forms of HO (30, 31). Furthermore, the sequence of histological events leading to the formation of the ectopic endochondral bone is similar in the two conditions. Accordingly, it has been suggested that HO in FOP and in non-genetic conditions might be mediated by common effectors and progenitor cells (34).

In the last years, several studies have focused on the cellular origin of ectopic ossification and potential candidates have shown osteogenic potential both *in vitro* and *in vivo* (34–37, 51, 53, 54). Endothelial involvement in induced and genetic HO is still actively debated (55–57). Early lineage tracing studies indicated Tie2-expressing vascular cells as the leading candidates for the cellular origin of BMP2-mediated heterotopic cartilage and bone (36). It has been recently shown that the main Tie2^+^ cells contributing to ectopic chondro-ossification in a transgenic model of FOP carrying the *ACVR1*^R206^ mutation are fibroadypogenic precursor cells (FAP) (57). This same study overruled the contribution of *bona fide* ECs to cartilage or bone formation on the basis of a VE-Cadherin dependent lineage tracing murine system, different from the one we used, first described in Alva et al. (58). In this transgenic line labeling efficiency in adult skeletal muscle has never been properly evaluated and quantified (39), therefore underestimation of EC contribution to endochondral ossification is possible.

By taking advantage of an efficient and endothelial-specific genetic lineage tracing mouse system (16, 38–40), we have followed the fate of ECs and their progeny in pathological chondro-osteogenesis leading to ectopic bone formation. We show here that EC-derived cells show the potential commitment to the chondrogenic lineage upon EndoMT induction in the skeletal muscle. Furthermore, upon BMP2 stimulation, muscle EC-derived cells express pSMAD1-5-8, indicating that the BMP pathway has been activated in these cells, and started to express Sox9 and Runx2, master factors of chondrogenesis (46), while downregulating endothelial markers, such as CD31.

EndoMT has been previously proposed to contribute to early phases of HO (51, 54, 59) and inhibition of EndoMT could rescue HO *in vivo* and *in vitro* (59). It has been suggested that the “transition” to a mesenchymal state in EndoMT and in Epithelial to mesenchymal transition (EMT), could indeed favor the acquisition of “stem-like” properties. While this has been fully demonstrated only for the process of (EMT) (60), it could account for many observation regarding ECs, including the co-expression of stem, endothelial and mesenchymal marker and their plasticity to differentiate to various lineages in both physiological and pathological conditions (18, 61). Some of the EC-derived cells derived cells expressing Sox9 are still proliferating, indicating that it is not a direct endothelial to chondrocyte conversion, but the process may require a step through a stem/progentitor state.

Another aspect to be considered when evaluating and comparing the EC-derived cells contribution to induced and genetic HO is the presence of the recurrent *ACVR1*^R206H^ mutation. It is quite predictable that mutation in the *ACVR1* (alias *ALK2*) locus would greatly impact the response and fate of all potential chondro-osteogenic progenitors. Indeed, iPS derived EC from FOP patients showed increased SMAD1/5/8 signaling and early chondro-osteogenic differentiation upon BMP4 stimulation (chondrocytes and immature osteoblasts), even if they could not differentiate in mature osteoblasts (20).

Our results indicate that in a “wild-type” environment, BMP activation can convert a number of “wild-type” EC-derived cells in chondrogenic precursors cells, that, regardless of whether they are bipotent osteo-chondroprogenitors or not, can contribute to the process of ectopic endochondral ossification (62).

Notably we show that EC-derived cell differentiation toward a chondrogenic lineage increases if the inflammatory environment is affected, and this is followed by an exacerbated ectopic bone formation.

The role of cells of the innate and adaptive immune system of in physiological ossification and HO has always been regarded as crucial (63–65). A particular focus has been set on the role of MPs, since they play a crucial function in tissue remodeling (13). A generalization of what is *in vivo* a broad and partially *continuum* set of differentiating populations suggests that activated MPs generate M1 (“classically activated”) and M2 (“alternatively activated”) cells (66). M1 macrophages are mainly involved in the response against pathogens, while M2 macrophages in the later phases of inflammation, and tissue regeneration and adaptation (66, 67). M2 cells play also angiogenic and vascular protective roles in inflamed tissues (66, 67). Indeed, we and others have demonstrated that MPs are necessary to orchestrate proper tissue remodeling and repair upon muscle injury, also by favoring angiogenesis, via the production of several secreted mediators and counteracting EndoMT (16, 68).

However, MP role might be more complex since it is becoming clearer that MPs are far more heterogeneous than what predicted before (69). It has been demonstrated for example that depletion of differentiated macrophages promoted an osteogenic environment whereas depletion of early lineage macrophages resulted in osteopenia (70, 71). Phagocyte/monocyte depletion was also found to counteract ectopic ossifications in models of Neurological HO and FOP (48, 49, 72). Still the characterization of remaining phagocytes in the soft tissues where HO was occurring has never been performed in these latter studies, therefore it is not clear what was the depletion magnitude *in situ* and which populations were depleted.

In our model not only we can achieve a 50–60% depletion of circulating CD11b^+^/F4/80^+^ monocytes in agreement with our previous findings (16, 40), but we can also show a 2–3 fold decrease in the number of F4/80^+^ macrophages infiltrating the muscle upon CLL treatment. Furthermore, we show that that amongst the depleted cells many are alternatively activated macrophages, e.g., the ones recognized to play a role in supporting proper muscle healing and angiogenesis (67).

It has been shown that in a model of NHO induced by spinal cord injury, that triggers a massive infiltration of inflammatory monocytes (Ly6C^high^), CLL treatment could lead to increased HO (73). Conversely, depletion of infiltrating macrophages, in particular of alternatively activated populations, in a BMP2/injury HO model, correlated with an increased EndoMT, increased chondrogenesis of EC precursors, and exacerbated bone lesion. It is conceivable that these apparently discrepant finding, may result from a different immune environment at the ossification site due to the different HO trigger and consequently, the different effect of the depletion, and also others phagocytic cells may be depleted with this approach.

CLL treatment lead to sustained BMP signaling. Despite the fact that we cannot univocally pinpoint the cellular source of BMP, this enhanced signaling may correlate with an increase in expression of hypoxia inducing factors as also shown before (16). This is also in agreement with previous work demonstrating that hypoxia is a promoting factor for HO, through amplification of BMP signaling (50). Indeed, we were able to partially inhibit HO in our model system by administration of the ACVR1/BMP/SMAD inhibitor dipyridamole, confirming that sustained BMP signaling plays a key role inducing HO in our monocyte depleted model.

Further studies will be necessary to get more insight in the signaling events underlying the crosstalk between the different osteoprogenitor cells, macrophages and other immune cells remaining in the osteogenic niche after depletion. This would allow the identification of agents that selectively sustain those immune cells that directly or indirectly protect from acquired and hereditary HO, as a more valuable strategy with respect to a general anti-inflammatory approach.

## Materials and Methods

### Animals

Mice were housed in the SPF facility at San Raffaele Scientific Institute (Milan, Italy) and treated with the approval of the Institutional Animal Care and Use Committee (IACUC 489, 663). Cdh5-CreER^T2^ (74) and R26R-EYFP (75) or tdTomato (76) mice were bred to yield heterozygous siblings and genotyped as in Wang et al. (74). Cre recombination was induced in Cdh5-CreER^T2^:R26R-EYFP or Cdh5-CreER^T2^: tdTomato mice at post-natal days 6-7-8 with three subcutaneous injections of Tamoxifen (250 μg/mouse; Sigma-Aldrich, St. Louis, MO, USA).

### Depletion of Circulating Phagocytes

Three months old Cdh5-CreER^T2^:R26R-EYFP or Cdh5-CreER^T2^: tdTomato mice were injected intravenously (i.v.) with liposomes containing either clodronate (CLL; 1.8 mg/mouse) or PBS (Sham) (http://www.clodronateliposomes.org/ashwindigital.asp?docid=26). The treatment was performed 1 day before cardiotoxin (CTX) injection (5 μl CTX 100 μM, from *Naja mossambica mossambica*, Sigma-Aldrich, Buchs, SG, Switzerland) and every 3 days afterward (at 2, 5, 8, 11, 14, 17, and 20 days after CTX injection).

### Flow Cytometry

EYFP^+^ and Tomato^+^ EC-derived cells from Cdh5-CreER^T2^:R26R-EYFP and Cdh5-CreERT2:tdTomato mice, respectively, were isolated from adult mice after muscle dissection. Tissues were cut in small pieces and dissociated with 0.15 mg/ml Collagenase IV (Roche, Basel, Switzerland) and 0.25% Trypsin (Gibco, Thermo Fisher Scientific, Waltham, MA, USA) or 0.4 mg/ml Dispase (Gibco). Dissociation reaction was performed at 37°C for 30 min for 2–3 cycles. Resuspended mononucleated cells were filtered with 70 and 40 μM filters. Cells were suspended in either DMEM with 20% FBS, 20 mM HEPES, 2 mM EDTA or PBS with 2% FBS, 2 mM EDTA (for antibody staining). For FACS analyses on circulating cells, blood was retrieved from the mouse tail, washed with red lysis buffer and incubated at 4°C for 30 min in blocking solution (PBS with 2% FBS, 2 mM EDTA). Cell sorting was performed using the MoFLoXDP (Beckman Coulter, Inc., Brea, CA, USA) or FACSAria Fusion (BD Biosciences, Bedford, MA, USA). FACS analysis was carried out using the BD FacsCANTO II (BD BioscienceUSA). Data were analyzed by FlowJo (TreeStar) and/or FCS Express 6 (De Novo Software, Los Angeles, CA, USA). The antibodies used are listed in Supplementary Table S3.

### Histochemistry, Immunohistochemistry, and Immunofluorescences

Serial muscle sections were stained with H&E (Sigma-Aldrich), according to standard procedures. Muscle sections were stained with H&E or Masson Trichrome (Bio-Optica, Milan, Italy), according to the manufacturers' instructions.

Immunohistochemistry (IHC) was performed on muscle frozen sections fixed with 4% PFA treated with 0.3% H_2_O_2_ and with an avidin-biotin blocking kit (Vector Laboratories, Burlingame, CA, USA), according to the manufacturer's instructions. Sections were blocked with 5% BSA, 0.1% Triton and 10% donkey serum in PBS for 1 h at RT. Subsequently, sections were incubated O/N with primary antibody. Primary Ab was revealed using biotin-conjugated anti-rat (1:300) IgG (eBiosciences, San Diego, CA, USA) and HRP streptavidin (Vector Laboratories), and detected using Vector NovaRED substrate kit (Vector Laboratories). Specimens were counterstained with DAPI (Molecular Probes, Life Technologies, Carlsbad, CA, USA) and examined with a Nikon Eclipse 55i microscope (Nikon, Tokyo, Japan). Immunofluorescence (IF) on frozen section was carried out as in Zordan et al. (16). The antibodies used are listed in Supplementary Table S4. Images were acquired using the following microscopes: Leica TCS SP2 Laser Scanning Confocal or Zeiss LSM 710 Confocal Microscope. Images were processed using Adobe Photoshop CS6 and Adobe Illustrator CS6. For quantification of chondroosteogenic cells, at least 7 fields were acquired for sample, cells positive for a chondrogenic or osteogenic markers (Sox9, Runx2, Osterix, SMAD1-5-8) and cells co-expressing reporter gene and chondrogenic or osteogenic markers were counted. For quantification of macrophages, at least 6 fields were acquired for sample, cells positive for an F4/80 and cells co-expressing CD163 or CD206 were counted. DAPI negative cells were excluded from quantification.

### Transcriptome Sequencing (RNA-seq)

Total RNA samples were extracted from freshly sorted EYFP^+^ EC derived cells of muscle of CLL- and Sham-treated mice using ReliaPrep^TM^ RNA Cell Miniprep System (Promega, Milan, Italy), checked for integrity on 2200 TapeStation instrument (Agilent Technologies, Santa Clara, CA, USA) and stored at −80° until use. Starting from 200 ng total RNA, RNA-seq libraries were prepared using the Illumina TruSeq Stranded mRNA Library Prep Kit (Illumina, San Diego, CA, USA), according to manufacturers' instructions, and sequenced on MiSeq platform (Illumina) in 76-cycle paired-end runs. Three independent replicates were sequenced for each condition (CLL and Sham). Raw sequence data are available in NCBI Short Reads Archive (SRA) under Accession Number PRJNA471032.

After fastq quality control by using FastQC tool, raw reads were mapped to the mouse reference genome (Mus musculus UCSC mm10/GRCm38) using STAR aligner (v.2.3.1s) and gene counts were calculated by HTSeq (v.0.6.1), using the Gencode M12 GTF file as gene model.

Differential gene expression analysis was carried out using DESeq2 software (v.1.0.17) to perform a pairwise comparison between CLL vs. Sham replicates. Low expressed genes (mean normalized count across all samples <10) were filtered out before testing genes for statistical significance. A False Discovery Rate [FDR, Benjamini and Hochberg (BH) correction] <0.05 was used as cut-off to define statistically significant differentially expressed genes (DEGs) in CLL vs. Sham samples. DEGs with log2 fold change > 0 were flagged as up-regulated, while genes with log2 fold change <0 were flagged as down-regulated in CLL vs. Sham samples.

ToppGene suite was used to perform gene enrichment analysis of statistically significant DEGs for Gene Ontology (GO) categories and pathways (https://toppgene.cchmc.org/enrichment.jsp). An FDR (BH correction) <0.05 was applied to all the annotation terms to defined statistically significant enrichment.

### Quantitative Real-Time PCR

Reverse transcription (RT) was done using the High-Capacity cDNA Reverse Transcription Kit (Applied Biosystems, Foster City, CA, USA). qRT-PCR analysis was carried out using the LightCycler 480 Instrument (Roche) or the 7900HT FAST Real-Time PCR detection system (Applied Biosystems). cDNAs were amplified using the GoTaq qPCR Master Mix and the Hot Start Polymerase (Promega). Primer sequences are listed in Supplementary Table S5. Ct values >35 were considered as negative. Each data points for every biological replicate was analyzed in triplicate. Quantification was performed using the relative ΔCt method. 28S or cyclophilin A genes were used as internal controls.

### *In vivo* Heterotopic Ossification

0.1 μg/μl of rhBMP2 (Peprotech, Rocky Hill, NJ, USA) in 100 μl growth factor-reduced Matrigel (BD Biosciences, 1:100 dilution) were injected intramuscularly in the quadriceps of Cdh5-CreER^T2^:R26R-EYFP or Cdh5-CreER^T2^: tdTomato 3-month-old mice. The contralateral muscle was used as internal control and injected with Matrigel only. Both quadriceps muscles were injected with 5 μl CTX 100 μM (from *Naja mossambica mossambica*, Sigma-Aldrich, Buchs, SG, Switzerland) to increase muscle damage. Animals were anesthetized by inhalation of 2-bromo-2-chloro-1,1,1-trifluoroethane, ≥99% (Sigma-Aldrich) before the injection.

### *In vivo* Micro-Computerized Tomography Imaging of Heterotopic Ossification

At day 10, 21 and 30 after BMP injection, *in vivo* micro-computerized tomography (μCT) scans were carried out to assess progression of ossification and any other effect on the normal skeletal structure. *In vivo* μCT imaging was performed using the IVIS SpectrumCT Pre-clinical *in Vivo* Imaging System (Perkin-Elmer, Waltham, MA, USA). μCT images were acquired without any contrast medium, with the following parameters: x-ray tube voltage = 50 kV, tube current = 1 mA, x-ray focal spot size = 50 μm. The μCT images calibrated in Hounsfield unit (HU) were reconstructed with a voxel size of 75 μm^3^. Threshold-based image segmentation was performed to obtain a 3D reconstruction and quantification of the ossification.

The total mineralized volume V = N × voxel size (mm^3^) was quantified using MIPAV (Medical Image Processing Analysis and Visualization) and MATLAB software, where N is the number of voxels corresponding to bone derived from the image segmentation procedure. The bone density (BD) quantification was calculated using the following formula:

(1) $$\begin{array}{r} BD=\frac{\sum_{i=0}^{N}HU_{i}}{V} \end{array}$$

The IVIS SpectrumCT was also used to obtain 3D fluorescence images using a transillumination excitation source placed below the animal. More precisely, a set of 2D fluorescence images were acquired at different transillumination points within the region of interest. 3D images were then reconstructed using the Fluorescence Imaging Tomography (FLIT) algorithm, as described in Kuo et al. (77), and implemented in the Living Image 4.5 software (Perkin Elmer). Transillumination fluorescence images were acquired using the following setting: excitation filter = 570 nm, emission filter = 620 nm, f-stop = 2, camera binning = 8, exposure = auto, field of view = 13 cm.

### Dipyridamole Treatment

Ten milligram per kilogram (body weight) dipyridamole was intraperitoneally (i.p.) administered daily to the treated animals in a solution composed of 10% ethanol, 5% 2-pyrolidone, 12–15% propylene glycol, 10% Cremophor ELP, saline to 100% as in Cappato et al. (47).

### Statistical Analysis

The number of animals needed to obtain statistically reliable data was calculated on the basis on published previous work carried out in our laboratory (16, 47) to minimize the number of animals used avoiding both an excess of animals used for each experiment and unnecessary repetitions and ensuring that each experiment produces meaningful data according to the principles of the 3Rs (Replacement, Reduction, and Refinement). The subjects allow to highlight differences between groups as regards the differences in macrophage depletion, gene expression, EC-cell counting and contribution to the various lineages of at least one standard deviation, with a power of at least 80% (error alpha = 0.05). Data were analyzed with Microsoft Excel 14.1.0 and GraphPad Prism 6 and were plotted as mean ± standard deviation (SD) or mean ± standard error of mean (SEM) or mean and min/max values. To evaluate statistical significance, unpaired two-tailed Student‘s *t*- tests were used assuming equal variance. Differences among three different experimental groups were evaluated by ANOVA analysis with Bonferroni as *post-hoc* tests.

## Data Availability

The datasets generated for this study can be found in NCBI Short Reads Archive, PRJNA471032.

## Ethics Statement

This study on mice was carried out in accordance with the European Community guidelines, the recommendations of San Raffaele Institute and University of Milano Bicocca Institutional Animal Care and Use Committees and the authorization by Italian Ministry of Health (no. 489, 663).

## Author Contributions

All contributing authors have agreed to submission of this manuscript for publication. MT designed and carried out most of the experiments and analyzed data. AV, MS, and PZ carried out some of the immunohistological experiments and analyzed data. AG and PN designed and carried out some of the gene expression analysis experiments. AG, AS, and CG designed and carried out the *in vivo* imaging experiments and analyzed data. IC designed and carried out the NGS experiments and analyzed data. RM, RB, and RR discussed results and provided important advice on experimental design. SB designed experiments, analyzed and interpreted data, directed the project, and wrote the manuscript with comments from all authors.

### Conflict of Interest Statement

The authors declare that the research was conducted in the absence of any commercial or financial relationships that could be construed as a potential conflict of interest.

## Supplementary Material

Movie S1 3D μCT reconstruction of the HO in BMP2/Sham treated Cdh-5CreERT2: tdTomato mice, 21 days after BMP2 injection. The 3D reconstruction is representative of at least three independent experiments.

Movie S2 3D μCT reconstruction of the HO in BMP2/CLL treated Cdh-5CreERT2: tdTomato mice 21 days after BMP2 injection. The 3D reconstruction is representative of at least three independent experiments.

Movie S3 3D FLIT (fluorescence imaging tomography) reconstructions and quantification of fluorescent signal at source (HO) of CLL-treated Cdh-5CreERT2: tdTomato mice. The 3D reconstruction is representative of at least three independent experiments.
