# Supplementary material for: Severe Heterotopic Ossification in the Skeletal Muscle and Endothelial Cells Recruitment to Chondrogenesis Are Enhanced by Monocyte/Macrophage Depletion
Source: Front Immunol. 2019 Jul 19;10:1640. doi: 10.3389/fimmu.2019.01640 (PMC6662553; doi:10.3389/fimmu.2019.01640)
Supplement: Supplementary file 2 [file Table_2.DOCX]

**Table S2**. **Percentage of endothelial derived cells expressing chondrogenic markers or BMP signaling pathway molecules in the quadriceps of Cdh5-CreER^T2^:R26R-EYFP mice 7 days after ectopic bone induction.**

| **EYFP/Runx2** | | **EYFP/Sox9** | | **EYFP/pSmad1/5/8** | |
| --- | --- | --- | --- | --- | --- |
| Mean | SEM | Mean | SEM | Mean | SEM |
| 1,22 % | ± 0,61 | 1,41 % | ± 0,34 | 2,88 % | ± 2,14 |
