# Supplementary material for: Severe Heterotopic Ossification in the Skeletal Muscle and Endothelial Cells Recruitment to Chondrogenesis Are Enhanced by Monocyte/Macrophage Depletion
Source: Front Immunol. 2019 Jul 19;10:1640. doi: 10.3389/fimmu.2019.01640 (PMC6662553; doi:10.3389/fimmu.2019.01640)
Supplement: Supplementary file 3 [file Table_3.DOCX]

**Table S3. Antibodies used for FACS analysis.**

| **Primary Antibodies and Conjugates** | | | | |
| --- | --- | --- | --- | --- |
| **Antibody** | **Host** | **Dilution** | **Clone** | **Supplier** |
| F4/80-APC | Rat | 1:100 | CI:A3-1 | Abd Serotec |
| CD45-PE | Rat | 1:100 | 30-F11 | BD |
| CD11b-PeCy7 | Mouse | 1:100 | M1/70 | BD |
| CD31-APC | Rat | 1:100 | 390 | EBioscience |
| CD31-PE | Rat | 1:100 | 390 | EBioscience |
| CD140B | Rat | 1:100 | APB5 | EBioscience |
| Alpha7 integrin | Mouse | 1:100 | 3C12 | MBL International |
