# Supplementary material for: Severe Heterotopic Ossification in the Skeletal Muscle and Endothelial Cells Recruitment to Chondrogenesis Are Enhanced by Monocyte/Macrophage Depletion
Source: Front Immunol. 2019 Jul 19;10:1640. doi: 10.3389/fimmu.2019.01640 (PMC6662553; doi:10.3389/fimmu.2019.01640)
Supplement: Supplementary file 4 [file Table_4.DOCX]

**Table S4. Antibodies used for IF analysis**

| **Primary Antibodies** | | | |
| --- | --- | --- | --- |
| **Antibody** | **Host** | **Dilution** | **Supplier** |
| CD31/PECAM1 (MEC13.3) | Rat | 1:2 | Gift from E.Dejana |
| GFP | Chicken | 1:500 | Abcam |
| p-SMAD1/5/8 (D5B10) | Rabbit | 1:800 | Cell Signaling |
| Osterix | Rabbit | 1:300 | Abcam |
| Sox9 | Rabbit | 1:300 | Millipore |
| Runx2 | Rabbit | 1:1600 | Cell Signaling |
| Ki67 | Rabbit | 1:100 | Biocare Medical |
| Osteocalcin | Rabbit | 1:300 | Abcam |
| F4/80 | Rat | 1:300 | Santa Cruz |
| CD163 | Rabbit | 1:200 | Santa Cruz |
| CD206 | Rat | 1:100 | Bio-Rad |
| **Secondary Antibodies** | | | |
| **Antibody** | **Host** | **Dilution** | **Supplier** |
| Anti-Rabbit Alexa 488 | Donkey | 1:500 | Molecular Probes |
| Anti-Rat Alexa 546 | Goat | 1:500 | Molecular Probes |
| Anti-Rat Alexa 647 | Chicken | 1:500 | Molecular Probes |
| Anti-Mouse Alexa 546 | Goat | 1:500 | Molecular Probes |
| Anti-Chicken Alexa 488 | Goat | 1:500 | Molecular Probes |
