# Supplementary material for: Severe Heterotopic Ossification in the Skeletal Muscle and Endothelial Cells Recruitment to Chondrogenesis Are Enhanced by Monocyte/Macrophage Depletion
Source: Front Immunol. 2019 Jul 19;10:1640. doi: 10.3389/fimmu.2019.01640 (PMC6662553; doi:10.3389/fimmu.2019.01640)
Supplement: Supplementary file 5 [file Table_5.DOCX]

**Table S5. Primers for qRT-PCR analysis**

| **Gene** | **Forward primer** | **Reverse primer** |
| --- | --- | --- |
| 28S | AAACTCTGGTGGAGGTCCGT | CTTACCAAAAGTGGCCCACTA |
| Cyclophillin A | CATACGGGTCCTGGCATCTTGTCC | TGGTGATCTTCTTGCTGGTCTTGC |
| PECAM1 | AGGGGACCAGCTGCACATTAGG | AGGCCGCTTCTCTTGACCACTT |
| Collagen 1 | GGTATGCTTGATCTGTATCTGC | AGTCCAGTTCTTCATTGCATT |
| Twist1 | GGACAAGCTGAGCAAGATTCA | CGGAGAAGGCGTAGCTGAG |
| Tcf4 | TCTGCAACTTCCCCTGACTT | TGTCTTGCAGGTTCTCATCG |
| Runx2 | GACTGTGGTTACCGTCATGGC | ACTTGGTTTTTCATAACAGCGGA |
| Sox9 | TCCAGCAAGAACAAGCCACA | CGAAGGGTCTCTTCTCGCTC |
| Osterix | ACCAGAAGCGACCACTTGAG | TTGGCTTCTTCTTCCCCGAC |
| Cadherin 5 | GTACAGCATCATGCAGGGCG | ATTCGTATCGGATAGTGGGG |
| FAP | TCAACTGTGATGGCAAGAGC | GTACCACATCGCCTGGAAAT |
| Bmp6 | ATGGCAGGACTGGATCATTGC | CCATCACAGTAGTTGGCAGCG |
| Bmp4 | TTCCTGGTAACCGAATGCTGA | CCTGAATCTCGGCGACTTTTT |
| Notch4 | ACGAGAGTACCACTCATTCGG | TAAGACCAATGTTGTCCTCGC |
| Jag2 | GACATCAATCCCAACGACTG | TAGGCGTCACACTGGAACTC |
| Flt4 | CTGGCAAATGGTTACTCCATGA | ACAACCCGTGTGTCTTCACTG |
| KlF4 | GTGCCCCGACTAACCGTTG | GTCGTTGAACTCCTCGGTCT |
| HI1-apha | TCAAGTCAGCAACGTGGAAG | TATCGAGGCTGTGTCGACTG |
