# Supplementary figures and images for: Severe Heterotopic Ossification in the Skeletal Muscle and Endothelial Cells Recruitment to Chondrogenesis Are Enhanced by Monocyte/Macrophage Depletion
Source: Front Immunol. 2019 Jul 19;10:1640. doi: 10.3389/fimmu.2019.01640 (PMC6662553; doi:10.3389/fimmu.2019.01640)

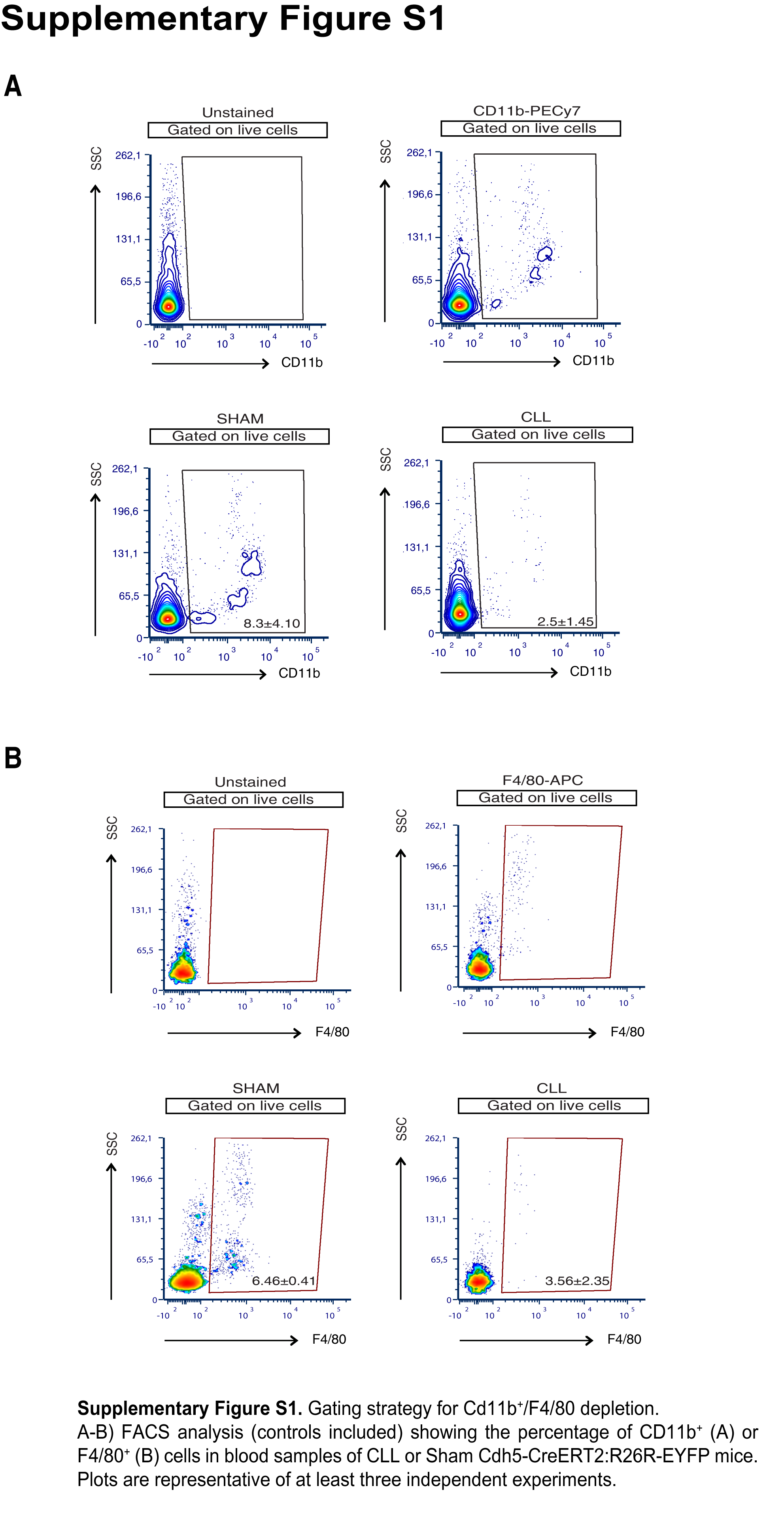

Supplement: Supplementary file 9 [file Image_1.tif]

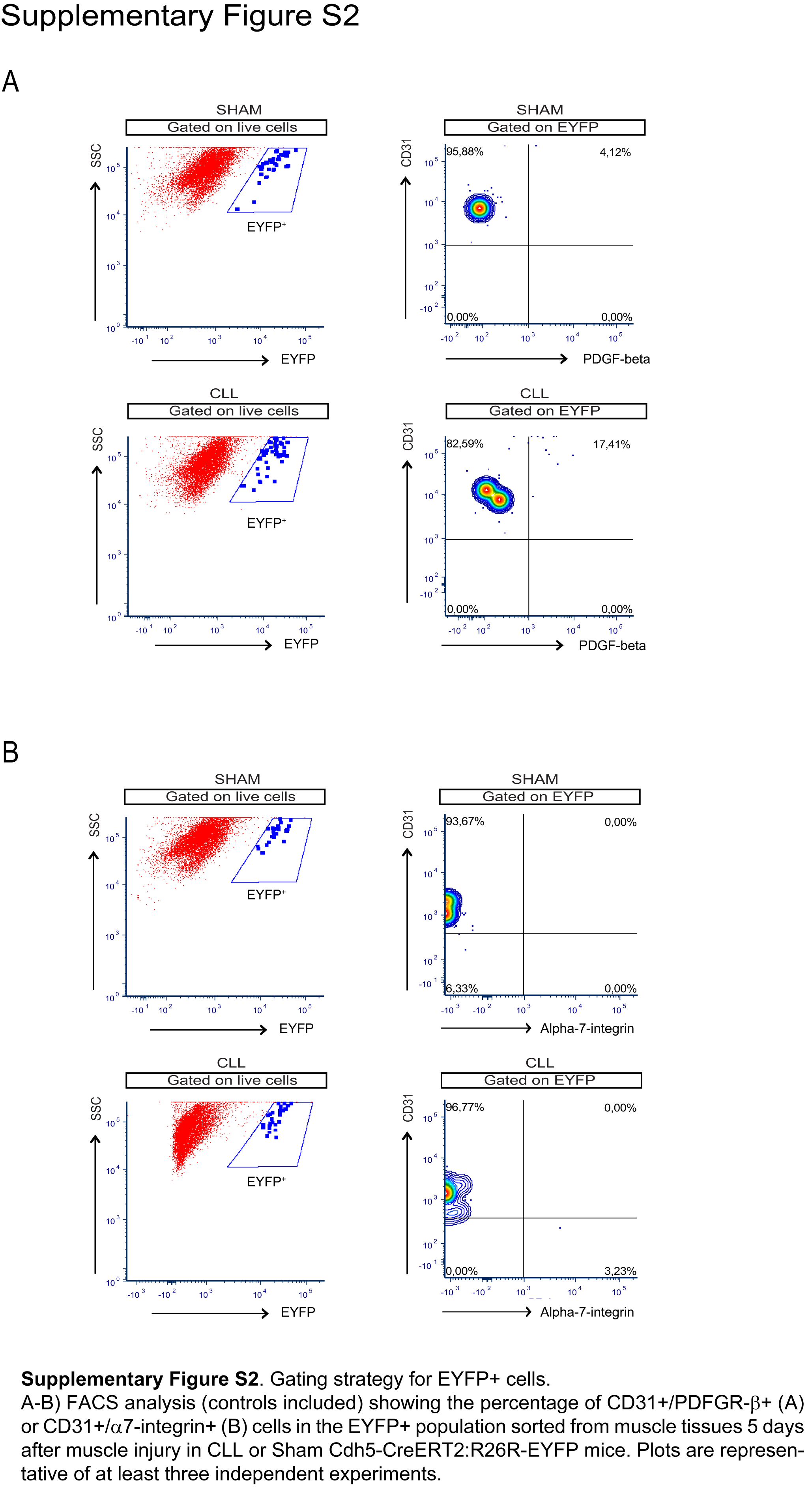

Supplement: Supplementary file 10 [file Image_2.tif]

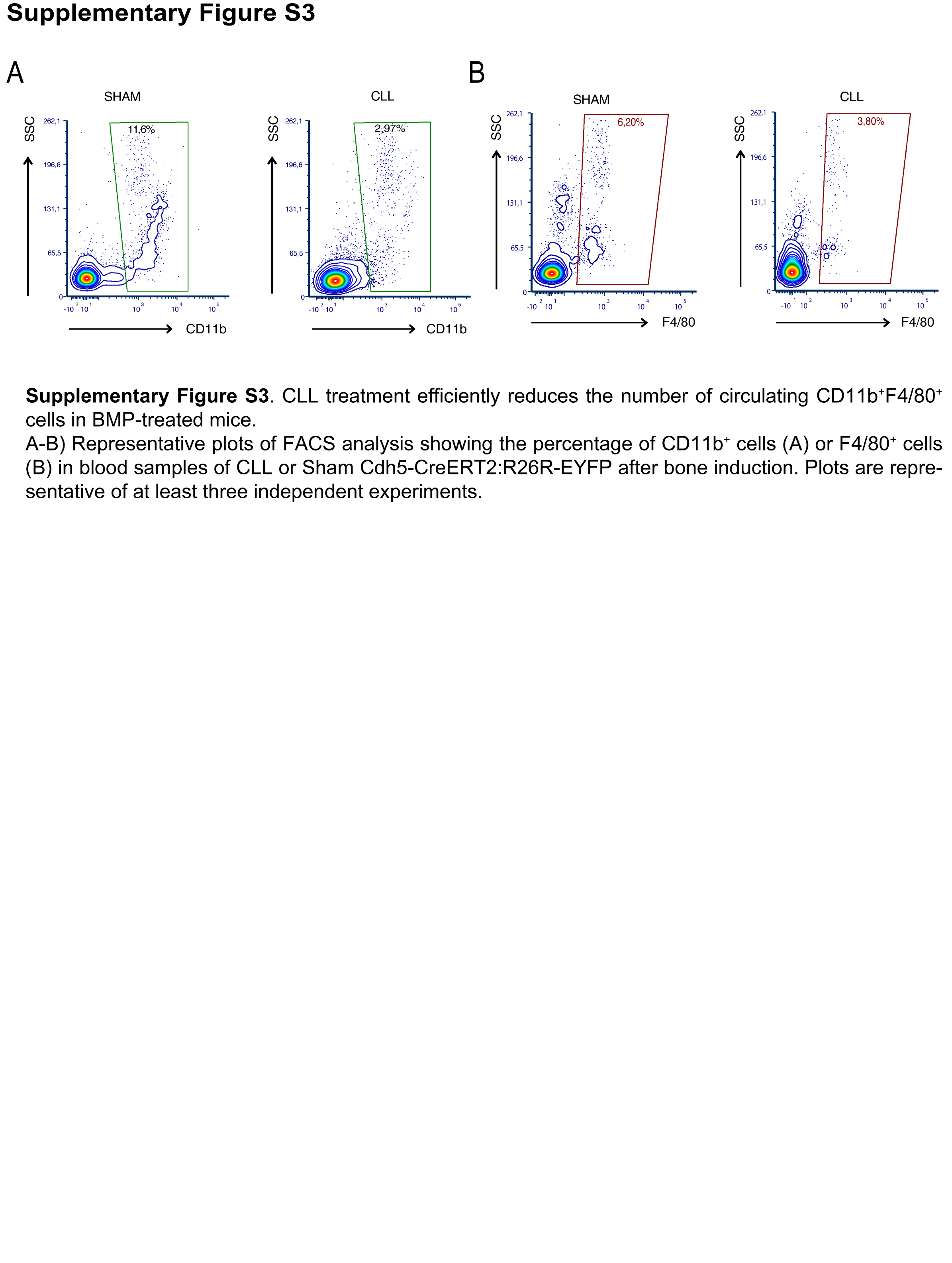

Supplement: Supplementary file 11 [file Image_3.tif]

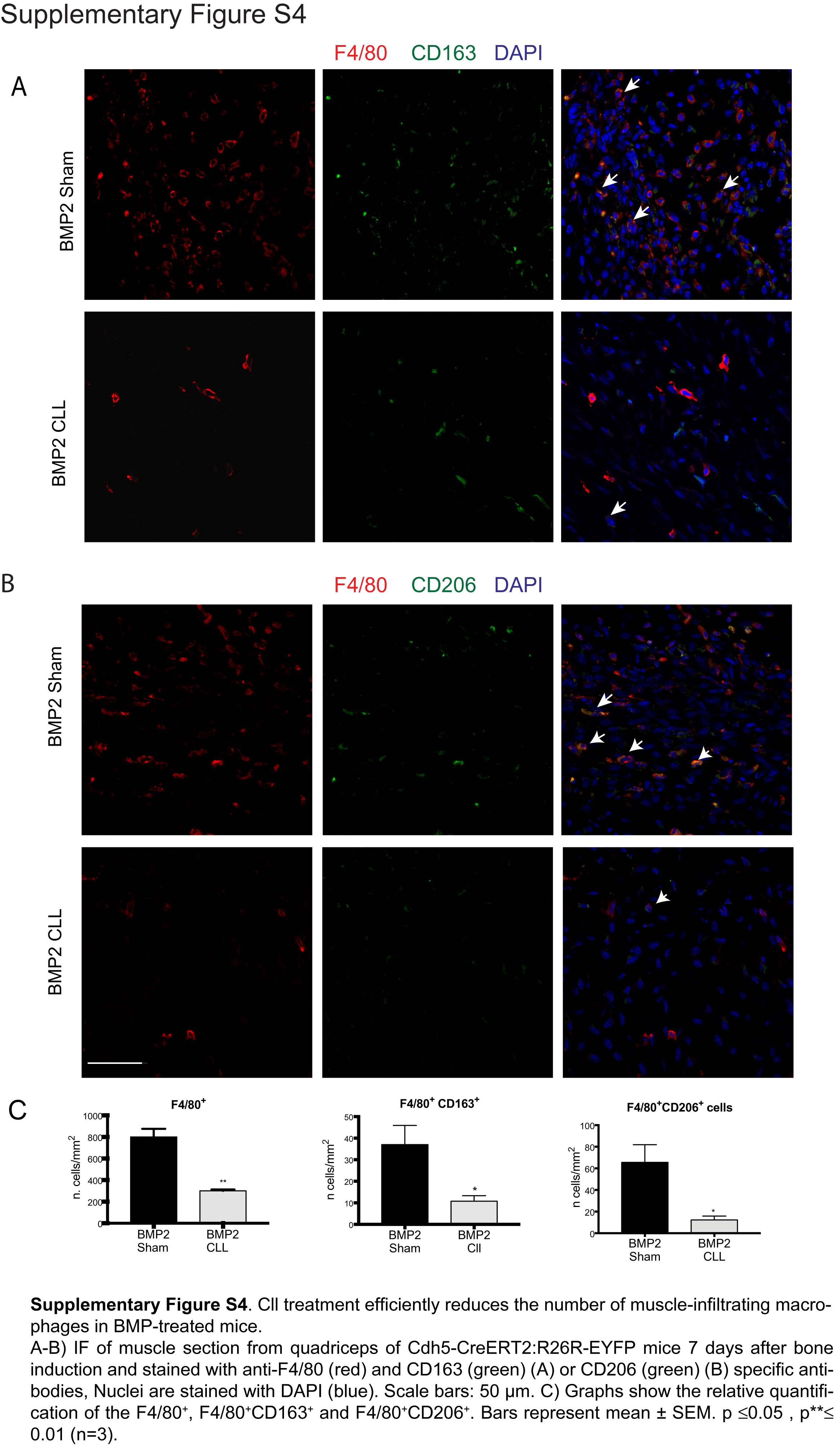

Supplement: Supplementary file 12 [file Image_4.tif]

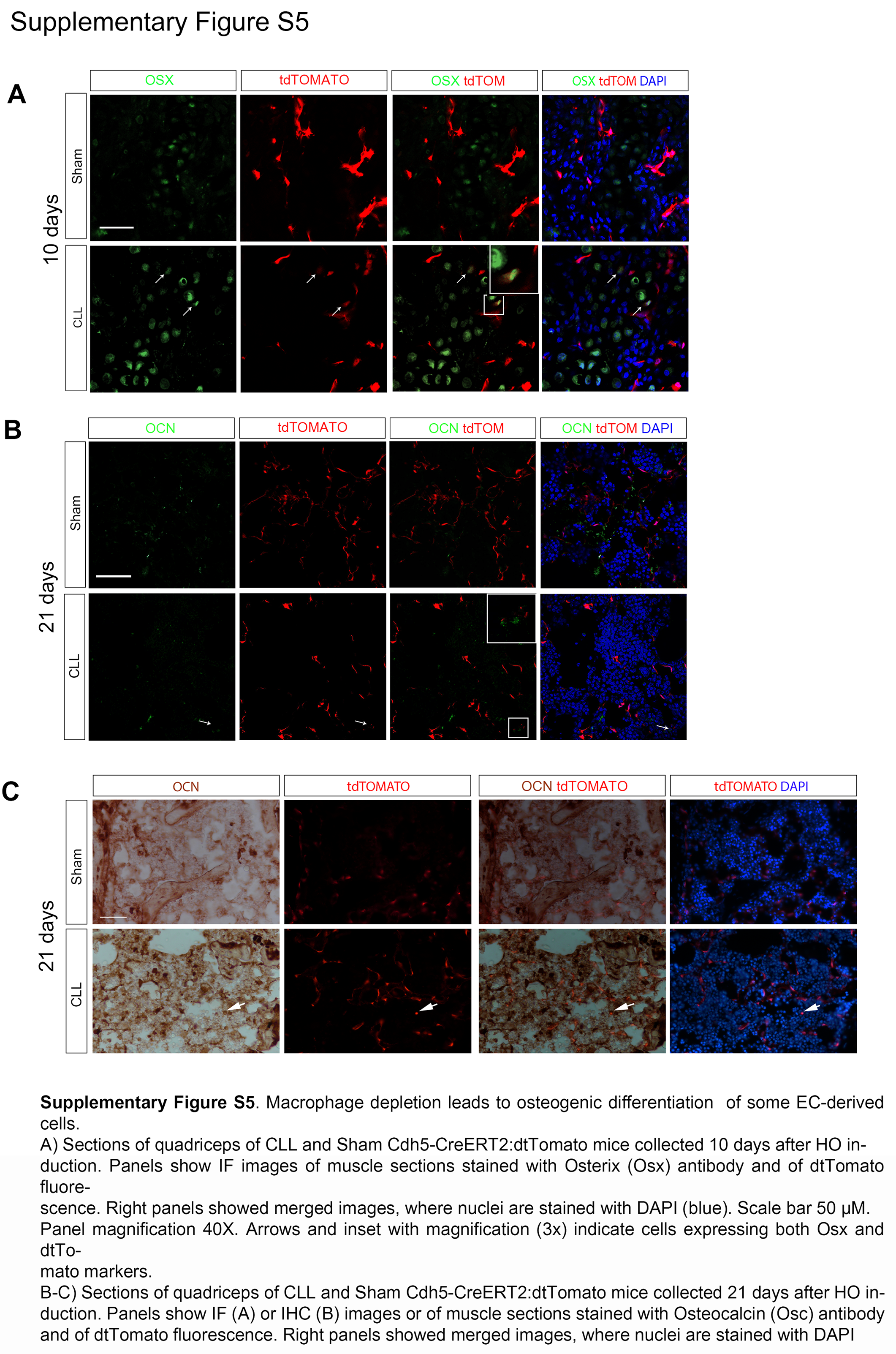

Supplement: Supplementary file 13 [file Image_5.tif]
